# Early Clinical Swallow Evaluation Is Associated with Lower Malnutrition Prevalence at Discharge in Neurogeriatric Rehabilitation Patients: A Prospective Study

**DOI:** 10.3390/nu18081288

**Published:** 2026-04-19

**Authors:** Enav Horowitz-Bor, Yulia Bugaevsky, Mona Boaz

**Affiliations:** 1 Department of Nutrition Sciences, Ariel University, Kiryat Hamada 3, Ramat Hagolan Street 36, Ariel 40700, Israel; 2 Bet-Rivka Geriatric Rehabilitation Center, Petah-Tikva 49245, Israel; yuliabu@clalit.org.il

**Keywords:** dysphagia, neurogeriatric, nutrition status, Mini Nutritional Assessment Tool Short Form

## Abstract

Background: Malnutrition is a known outcome of dysphagia. Objectives: To estimate the association between the timing of the Clinical Swallow Evaluation (CSE) at admission and nutrition status at hospital discharge. Methods: In this single-center prospective study, the electronic medical records of patients discharged from the neurogeriatric rehabilitation department at the participating hospital, who had undergone CSE by a speech pathologist per hospital bed-side evaluation protocol, were reviewed. The participants were divided into two groups: those who underwent CSE within 48 h of hospitalization per Israel Ministry of Health guidelines (early CSE, *N* = 31); and those who underwent CSE later than 48 h from admission (late CSE, *N* = 47). Nutrition status was evaluated by a registered dietitian using the Mini Nutritional Assessment Tool Short Form (MNA-SF). Nutrition status at discharge was categorized and compared between the groups. Results: Seventy-eight patients were included (mean age 80.7 ± 8.1 years); 85.9% were malnourished at admission, with no difference between groups. At discharge, malnutrition was less prevalent in the early vs. late CSE group (61.3% vs. 78.7%; *p* = 0.012). In multivariable logistic regression, late CSE increased odds of malnutrition at discharge by more than six-fold. Each additional year of age increased the odds of malnutrition by 13%; each additional point in baseline MNA SF score reduced the odds by 48%; and greater cognitive decline increased the odds of discharge malnutrition risk by more than 2.6-fold. Sex and the number of dietitian consultations were not associated with malnutrition at discharge. Conclusions: Early CSE in elderly patients hospitalized for neurogeriatric rehabilitation is associated with less malnutrition at discharge.

## 1. Background

Dysphagia can be defined as any difficulty in passing food from the oral cavity to the stomach [1]. The World Health Organization describes dysphagia as a “geriatric syndrome” and classifies it under “gastrointestinal symptoms and signs” [2]. Dysphagia is associated with important adverse outcomes including aspiration, decreased quality of life, dehydration, malnutrition, increased hospitalization days and higher mortality risk. Dysphagia prevalence increases in the presence of neurological conditions such as cardiovascular accident (CVA), Parkinson’s disease, head injuries, dementia and aging [2,3], but the prevalence rate in hospitalized adults aged ≥65 years is not precisely known. Prevalence estimates range from 25 to 85%, depending on the reason for hospitalization [3,4]. The worldwide population is aging, increasing the proportion of people at risk for dysphagia; therefore, it is essential to improve methods for screening, diagnosis and treatment. The importance of dysphagia among hospitalized adults has been emphasized by the joint committee of the European Society for Swallowing Disorders and the European Society for Geriatric Medicine (European Society for Swallowing Disorders) [4].

Malnutrition is a clinical condition characterized by an imbalance in energy, protein or other nutrients that manifests itself in negative effects on body size, composition and function, causing negative health outcomes [5]. Dysphagia significantly increases the risk for malnutrition [3,6]. In geriatric rehabilitation hospitals, malnutrition prevalence estimates range from 29 to 50% in studies from Europe and the USA [7]. Malnutrition among the elderly is associated with general functional decline, decreased muscle mass and strength (sarcopenia), and diminished quality of life, together with increased risk of falls, depression, and mortality [7].

Approximately 35% of patients aged 65 years or older experience a functional decline during an acute hospitalization [8]. The European consensus of the Society for Geriatric Medicine recommends geriatric rehabilitation for patients with geriatric syndromes and comorbidities and who have the potential to improve their functional performance [9].

A Clinical Swallow Evaluation (CSE) is performed by a speech therapist (ST) and aims to identify dysphagia or aspiration risk. Little information is available regarding CSE timing among geriatric rehabilitation patients, and its influence on nutrition treatment and outcomes. The present study is designed to examine whether such an association exists. Specifically, the primary objective of the present study is to assess and quantify an association between CSE timing at hospitalization onset and nutrition status (NS) as measured using the Mini Nutritional Assessment Short Form (MNA-SF) at the time of discharge from a neurogeriatric rehabilitation unit. It was hypothesized that patients who underwent CSE within the first 48 h from the time of admission would have greater MNA-SF [10] scores indicating better nutrition status at discharge than patients who underwent CSE later in the hospitalization. Additionally, the study aimed to develop a predictive model of nutrition status at discharge in which modifiable covariates could be identified. Identifying an association between CSE timing and malnutrition status at discharge would contribute to an evidence-based justification for early timing, which likely enlarges the nutrition therapy window, leading ultimately to improved nutrition and health outcomes.

## 2. Methods

### 2.1. Study Design

The present report is a prospective, single-center study. The data of all patients discharged from the neurogeriatric rehabilitation unit from January 2020 to October 2021 were extracted from electronic patient medical records. All extracted data were de-identified and could not be traced back to a specific participant.

All participants underwent a clinical swallowing evaluation (CSE), which was performed by a speech therapist (ST) using the “bed-side-evaluation protocol”, one of the methods recommended by the European Union Geriatric Medicine Society [4] and per Israel Ministry of Health guidelines [10]. CSE performance was classified according to the timing as early (CSE performed less than 48 h from the time of department admission, as specified by Israel Ministry of Health guidelines) or late (CSE performed 48 h or more from the time of department admission). Nutrition status assessment using the Mini Nutritional Assessment Tool Short Form (MNA-SF) was performed by a registered clinical dietitian, once during the first week from the date of hospital admission, according to Israel Ministry of Health guidelines [11], and once again at discharge, per department protocol.

### 2.2. Ethics

The present study was approved by the Helsinki Committee of the public geriatric rehabilitation center in Beit Rivka, located in Petah-Tikva, Israel: RMC-0495-21. Because all data were extracted from the electronic medical record and de-identified prior to inclusion in the data set, the study was granted exemption from requiring signed informed consent.

### 2.3. Study Population

Eligible for participation were patients hospitalized during the data acquisition period (1 September 2021–1 August 2022) aged 65 years or older and diagnosed with dysphagia during their rehabilitation hospitalization. All patients were admitted to the neurogeriatric rehabilitation unit for various reasons and the primary diagnosis was also varied. A total of 78 patients were eligible and included in the present study.

Excluded were patients not diagnosed with dysphagia on CSE; patients hospitalized for fewer than 20 days; patients who continued enteral feeding throughout their hospitalization; patients who underwent CSE or started swallowing rehabilitation treatments in a former unit of the same rehabilitation center, and were transferred for various reasons (epidemiological, change in status, etc.); patients missing anthropometric data; and patients who died during hospitalization.

The study sample size was calculated using the following assumptions: the MNA-SF category of malnutrition at the time of hospital discharge would be 50% in the early CSE group and 80% in the late CSE group, based on clinical observations. A sample size of 78 (*n* = 39 in each group) would provide the study with 80% power to detect a by-group difference of this magnitude with a two-sided alpha of 0.05.

### 2.4. Data Collection

The following data were extracted from the electronic medical record of each participant: demographic data excluding any identifying information including National Identity Number, name, date of birth; duration of hospitalization; comorbidities; primary reason for rehabilitation hospitalization; oral nutrition supplements (ONSs) use as part of nutrition support during hospitalization; and anthropometric measures including height, which was calculated per routine according to the ulna length index and body weight. Body weight was measured as part of the unit routine on scales adapted for mobility aids. Based on these data (height and weight), BMI (Body Mass Index) was calculated [12]. In addition, mobility data was collected according to motor Functional Independence Measure (FIM) scores per hospital routine. The FIM includes 13 motor items and 5 socio-cognitive items rated on a 7-point scale for each item, with higher scores indicating greater independence [13]. In order to assess cognitive-mental condition, the Mini Mental State Examination (MMSE) score was used. This test consists of 11 questions with a total maximum score of 30 points that screens for dementia and cognitive impairment by assessing the domains of orientation, memory, language, and attention. A score of 25 or greater is used to indicate no cognitive decline (normal function), 19–24 indicates mild decline, 10–19 moderate decline and 0–9 severe decline or impairment [14]. For the purpose of this study and in accordance with hospital protocol, the MMSE score was categorized as follows: greater or equal to 25 indicates no decline; 10–24 indicates some impairment; 0–9 indicates severe impairment. These were coded so that a higher score indicates greater impairment. The Geriatric Depression Scale (GDS-15) was used to assess patient depression. This 15-item version of yes/no questions is scored such that 0–4 is considered normal, 5–10 suggests mild to moderate depression, and 11–15 indicates severe depression [15]. Both the MMSE and GDS are performed routinely as part of the occupational therapy assessment. Prescribed medications were recorded.

### 2.5. Swallowing Assessment Procedure

CSE performance and timing were carried out in adherence with Israel Ministry of Health guidelines, according to which all patients admitted with an enteral feeding tube were evaluated in the first 48 h from the time of admission. If swallowing difficulties, either past or present, were recorded in the patient medical record, or if such history was brought to the attention of healthcare staff, CSE was ordered and performed during hospitalization, according to ST team scheduling. If a member of the healthcare team observed that a patient experienced an event of swallowing difficulty, such as coughing while eating or drinking [16], CSE was ordered and performed during hospitalization, according to ST team scheduling. Any of the aforementioned events triggered performance of the CSE using the “bed-side-evaluation protocol,” during which patients swallowed 50, 90 or 150 mL of water. An abnormal swallow was identified by coughing during or after the swallow, wet/hoarse voice quality, or slow swallow (<10 mL/s).

### 2.6. Nutrition Status Assessment

Nutrition status in the study population was evaluated using MNA-SF, a validated nutrition assessment tool [17] which consists of six domains: loss of appetite, weight loss, mobility status, acute illness or stress, neuropsychological status and BMI. In clinical practice, the MNA-SF scale of 0–14 points is divided into three categories, 0–7 points “malnourished”, 8–11 points “at risk of malnutrition”, 12–14 points “well-nourished,” such that a higher score indicates better nutrition status.

### 2.7. Outcome Measures

The primary endpoint of the present study is the correlation between the day on which the CSE was performed and the MNA-SF score at discharge. Secondary endpoints included a comparison of the change from baseline MNA-SF category by group (early vs. late CSE). Additionally, a regression analysis was used to identify predictors of improvement in NS.

### 2.8. Statistical Analysis

Statistical analyses were performed using SPSS V. 26.0 software (IBM, Armonk, NY, USA). Categorical variables are presented as *n* (%). Distributions of all continuous variables were tested for normality using the Kolmogorov–Smirnov test. Normally distributed continuous variables were described as mean ± standard deviation while those with distributions deviating from normal were described as median (interquartile range). Associations between nominal variables were assessed using the chi square test. By-group comparisons of continuous variables were conducted using the *t*-test for independent samples or the Mann–Whitney U as appropriate based on normality of distribution and variance heterogeneity. A multivariable logistic regression was performed to predict malnutrition at discharge in which the MNA-SF score was dichotomized into a new variable, “malnutrition,” such that an MNA-SF score of 0–7 indicated the presence of malnutrition (malnutrition = 1) and scores >7 indicated the absence of malnutrition (malnutrition = 0). Variable selection for this model was guided by (1) univariate analyses and (2) clinical experience. All tests were two-sided and considered significant if *p* < 0.05.

### 2.9. Potential Bias

Exposure was assessed using identical methods per Israel Ministry of Health guidelines in all participants regardless of CSE timing. Outcomes were also assessed using identical methods regardless of CSE timing. All demographic and medical variables were extracted from the patient electronic medical record. It is conceivable that recording, which was performed in the framework of a clinical rather than a research setting, was not uniform, potentially leading to misclassification of individuals in terms of exposure and/or outcome. However, such an instance would likely be non-differential, leading to an underestimate of the true association between CSE timing and nutrition outcomes. Finally, the prediction model of nutrition status score included potential confounders to enable estimation of the influence of CSE timing on this outcome.

## 3. Results

Data from 78 patients were included in the present study: 31 in the early CSE group, and 47 in the late CSE group. Table 1 presents baseline demographic and clinical characteristics of the 78 study participants, by CSE timing group. Participants were elderly (80.73 ± 8.10 years) and 51.3% were women. The main cause for rehabilitation was CVA (71.8%) and the majority (78.3%) of patients exhibited some level of cognitive impairment according to the categorized MMSE score at admission. Almost 30% of patients started their rehabilitation on enteral nutrition. Approximately 30% of patients were underweight per BMI category, while 85.9% were categorized as malnourished according to the MNS-SF. There were no significant differences between the treatment groups (early vs. late CSE) at baseline.

Table 2 describes treatment characteristics during rehabilitation. The average hospitalization was 60 days in duration. The overwhelming majority of patients (85.9%) received oral nutritional supplements (ONSs) daily, dosed according to their individualized nutrition care plan. Nutrition care sessions included frontal meetings between a registered dietitian and patients and/or family members; changing course of diet recommendations or ONS type; eating observation and intake tracking. On average, patients underwent six such sessions during their hospitalization, whereas they underwent 3.5 swallowing rehabilitation sessions, including observations, compensatory approach as viscosity and positioning modifications, swallowing exercises, and patient and family training sessions. There were no significant differences between the treatment groups in the number of nutrition or swallowing rehabilitation sessions undergone by patients. By definition, a significant difference in the variable “CSE day” was observed.

Table 3 presents nutrition status outcomes as well as cognitive status at discharge. A significant difference in the nutrition status of patients by MNA-SF category was observed. While discharge MNA-SF scores did not differ by CSE timing in univariate analysis, a significant association between CSE timing and MNA-SF scores at discharge was detected after controlling for continuous MNA-SF scores at admission: Discharge MNA-SF = 3.49 + (0.68 × admission MNA-SF, *p* < 0.001) − (0.93 × CSE timing, *p* = 0.04). In this equation, late CSE = 1 and early CSE = 0. The equation is significant (*p* < 0.001). Further adjustment for other covariates, including age, sex and cognition, reduced the strength of the association between CSE timing and discharge MNA-SF scores to marginal significance (*p* = 0.052).

**Figure 1:**
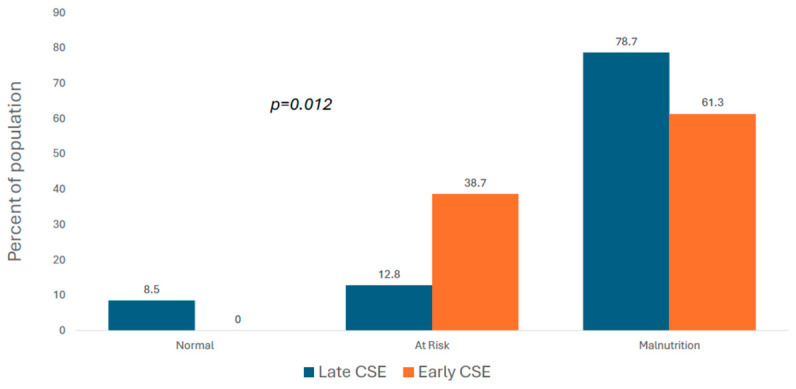
Nutrition status at discharge by clinical swallowing evaluation (CSE).

**Figure 2:**
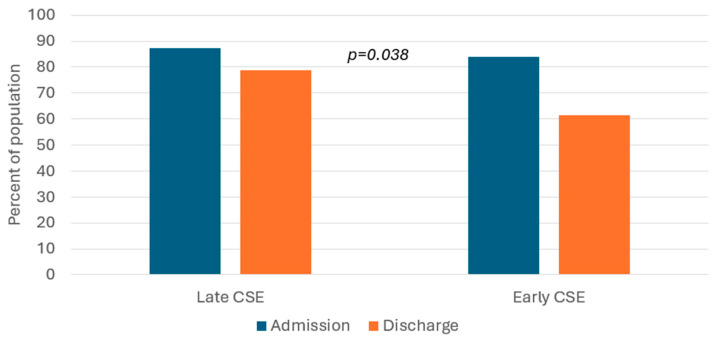
Change from baseline malnutrition category within clinical swallowing evaluation (CSE) group.

The percentage of patients with malnutrition at the end of follow-up was 17.4% lower in the early CSE group than in the late CSE group, as shown in Figure 1. Figure 2 presents the within-groups change in nutrition status category from baseline to the end of follow-up. The decrease in the percentage of patients in the malnourished category in the early CSE group was almost three times greater than the within-group decline in the late CSE group.

Table 4a presents the logistic regression model of malnutrition status as determined by the MNA-SF score at the end of follow-up. For this analysis the MNA-SF score was dichotomized into a new variable, “malnutrition,” such that an MNA-SF score of 0–7 indicated the presence of malnutrition (malnutrition = 1) and scores > 7 indicated the absence of malnutrition (malnutrition = 0).

The following variables were included in the model: CSE timing (early vs. late), indicating that compared to patients who received early CSE, those who received late CSE had more than six-fold odds of malnutrition at discharge; age, such that each additional year conferred an increase of 13% in the odds of malnutrition at discharge; MNA-SF score at hospital admission, such that each additional point reduced the odds of malnutrition at discharge by 48%; MMSE score at hospital admission (categorized so that 0 = normal cognition, 1 = cognitive deficit, and 2 = dementia) such that each step in declining cognition (each 1-point increase in score) was associated with an increase in the odds of malnutrition at hospital discharge of more than 2.6-fold. Sex and the number of sessions with a dietitian did not change the odds of malnutrition at the time of hospital discharge. The model was significant (*p* < 0.001) and correctly classified 84.6% of study participants, with 91.1% sensitivity and 68.2% specificity.

With a total of 56 events of malnutrition at discharge, the model had fewer than 10 events per variable (EPVs), which indicates an increased risk of over-fitting. When sex and number of sessions with a dietitian were removed from the model, odds ratios were essentially unchanged, but the model now had an EVP of 14, which indicates a low risk of over-fitting. This more compact model is presented in Table 4b. The model was significant (*p* < 0.001) and correctly classified 85.9% of study participants, with a sensitivity of 91.1% and specificity of 72.7%, indicating very little change from the larger model.

## 4. Discussion

The present study identified an association between CSE timing and MNA-SF-based nutrition status category at hospital discharge. Specifically, the odds of malnutrition at discharge, defined as an MNA-SF score of 0–7 points, was more than six-fold greater in the late vs. early CSE group after controlling for other variables including age, sex, MNA-SF score at hospital admission, MMSE score at hospital admission and the number of dietitian treatments received during hospitalization. Despite these findings, it must be emphasized that the study is cross-sectional, with a relatively small sample size and unequally sized exposure groups. These factors preclude any causal inference, and findings must be considered associative.

The finding that later assessment is independently associated with substantially higher odds of malnutrition at hospital discharge underlines the importance of early CSE in clinical settings. It has been suggested that early CSE might permit the immediate implementation of nutrition interventions individualized to specific patient needs including texture and viscosity adaptations and provide opportunities to initiate therapies promoting functional improvement [18,19]. Delays in CSE may miss feeding and swallowing problems that, left unmitigated, could contribute to worse nutrition outcomes. It has been suggested that early screening appears to confer a protective effect by enabling earlier nutrition and behavioral swallowing therapies and other interventions, leading to better nutrition outcomes [20].

Cognitive impairment was strongly associated with the odds of malnutrition at the time of hospital discharge. In this study, MMSE scores were categorized as follows: 0 = normal cognition; 1 = cognitive deficit; and 2 = dementia. Thus, a higher score reflected greater cognitive impairment, and each incremental decline in cognitive function markedly increased the odds of malnutrition. Dysphagia is a frequent finding in people with dementia [21], with a prevalence of up to 86% in hospitalized individuals with Alzheimer’s disease and other forms of dementia [22]. Cognitive impairment has been shown to be associated with increased odds of sarcopenia [23]. The association between dementia and dysphagia is thought to be bidirectional [21]. Feeding challenges faced by individuals with dementia include difficulties with meal initiation, a reduced ability to recognize hunger, impaired motor coordination required for self-feeding, and behaviors such as turning away from food, which, when combined with dysphagia, greatly increase malnutrition risk [17].

Nutrition status at the time of hospital admission was a robust predictor of later malnutrition. The MNA-SF score at admission remained independently protective, underscoring the utility of the MNA-SF in identifying patients at risk for malnutrition. But it must be restated that baseline MNA-SF scores did not differ by CSE timing, and CSE timing remained a strong predictor of discharge malnutrition independent of the MNA-SF score at admission. The continuous MNA-SF score did not differ at hospital discharge by CSE timing group; however, categorized MNA-SF nutrition status, the preferred measure [24], did differ by CSE timing group, such that late CSE strongly and independently predicted this outcome even after controlling for other variables. Further, in multivariable linear regression, CSE timing was significantly associated with the continuous MNA-SF score at discharge after controlling for baseline continuous MNA-SF score. Further adjustment mitigated the association between CSE timing and the continuous MNA-SF score so that it became only marginally significant (*p* = 0.052).

In the present study, malnutrition risk did not differ by sex. This finding contrasts with other reports, in which sex differences in malnutrition risk have been observed. In a study of older people with rheumatoid arthritis, malnutrition risk was a significantly more frequent finding in men than women [24]. By contrast, a study of malnutrition in geriatric syndromes found malnutrition prevalence was greater in females [25]. It is possible that these inconsistencies are attributable to the tool used to assess nutrition status and to the patient population studied and may suggest that malnutrition risk is driven more by clinical and functional factors than by biological sex differences.

The absence of an association between the number of nutrition therapy sessions and malnutrition status at discharge may be attributable to reverse causality [26]; patients who were more severely malnourished might have required additional sessions, thereby obscuring any potential benefit. Another possibility is that nutritional care itself is the meaningful exposure, without a clear dose–response relationship. Taken together, the lack of correlation between session frequency and discharge malnutrition—alongside the strong association between CSE timing and nutrition outcomes—suggests that early dysphagia assessment extends the overall duration of nutrition intervention, regardless of how many dietitian sessions a patient ultimately receives.

The present study has several strengths, including data extraction from validated tools used in clinical practice such as the MNA-SF and the MMSE. Additionally, the data were acquired in a “real life” setting in a neurogeriatric rehabilitation facility.

The findings of the study should be interpreted in light of several important limitations. The 48 h cutoff used to define early versus late CSE was based on Israel Ministry of Health guidelines. Although no differences in baseline characteristics by CSE timing were observed (Table 1 and Table 2), this does not preclude the presence of unmeasured differences. CSE timing may reflect underlying patient characteristics, such as medical complexity or non-communicable disease comorbidities such as diabetes, that could independently influence malnutrition risk at discharge. Additionally, because the study was conducted at a single center, generalizability may be limited. Delayed CSE may have resulted from factors such as impaired consciousness, which would be expected to affect dietary intake and nutritional outcomes, or from logistical factors such as weekend admission, when allied health staffing may be reduced [27]. Accordingly, caution is warranted in attributing a causal relationship to CSE timing, as it may serve as a proxy for other patient- or system-level factors.

Another limitation is the timing of the study itself, which commenced during the COVID-19 pandemic. This may have altered the typical patient population composition, skewing it toward the more severely ill [28]. It also brings into question whether findings from this time period are generalizable to non-pandemic periods.

While the study included the number of patients calculated as necessary to detect the primary study endpoint (*n* = 78), the distribution was not identical by CSE timing group. Specifically, there were 31 patients in the early CSE group and 47 in the late CSE group, a relative difference of 34%. This unbalanced distribution may have reduced study power to identify by-group differences [29]. On the other hand, while only marginally significant in the univariate comparison of malnutrition at discharge by CSE timing group, this endpoint was significantly predicted by CSE timing group after controlling for potential confounders.

## 5. Conclusions

Despite its limitations, the present study suggests an association between early CSE in hospital and rehabilitation settings and reduced malnutrition risk at discharge. Early CSE permits immediate nutrition intervention, increasing therapeutic exposure and potentially improving nutrition outcomes. Due to the predictive value of advanced age, malnutrition at admission and cognitive decline, it may be appropriate to enhance vigilance for patients in those categories. Nevertheless, it must be reiterated that the findings herein are cross-sectional, derived from a convenience sample of participants from a single neurogeriatric facility, and that assignment to exposure was not random. Thus, conclusions must be considered provisional and no causality can be inferred.

Future research should use larger patient populations to enable subgroup analyses by cognitive status, comorbidities and perhaps medication regimens. Further, clinical trials can establish an evidence base for optimal CSE and nutrition intervention timing. Overall, early identification and intervention remain important strategies for improving nutrition and clinical outcomes in hospitalized and rehabilitation populations.

## Figures and Tables

**Table 1:** Baseline patient data.

	All (*n* = 78)	Early CSE (*n* = 31)	Late CSE (*n* = 47)	*p* Value
Age (years)	80.73 ± 8.10	80.52 ± 7.91	80.87 ± 8.30	0.851
Gender (% Female)	51.3	61.3	44.7	0.151
**Main cause for rehabilitation (%)**				
CVA	71.8	71.0	72.3	0.895
Other	28.2	29.0	27.7	
**Cognitive status categories per MMSE (%)**				
Severe impairment (score = 0–9)	57.7	54.8	59.6	0.514
Mild/moderate impairment (score = 10–24)	20.5	25.8	17.0	
No impairment (score ≥ 25)	21.8	19.4	23.4	
**Enteral nutrition (%)**	23.1	32.3	17.0	0.118
**Body Mass Index (BMI)**	25.64 ± 5.53	24.86 ± 4.41	26.15 ± 6.14	0.423
**BMI** **categories** **(%)**				
Underweight (BMI < 18 kg/m^2^)	27.8	32.2	25.5	0.879
Normal (BMI ≥ 18–<25 kg/m^2^)	57.0	51.6	61.7	
Overweight (BMI ≥ 25 kg/m^2^)	14.1	16.1	12.7	
**MNA-SF score**	4.90 ± 2.23	4.74 ± 2.16	5.00 ± 2.29	0.694
**Nutrition status categories (%)**				
Malnutrition (score = 0–7)	85.9	83.9	87.2	0.570
At risk (score = 8–11)	12.8	16.1	10.6	
Normal status (score = 12–14)	1.3	0	2.1	

MMSE = Mini Mental State Examination; MNA-SF = Mini Nutritional Assessment Short Form.

**Table 2:** Rehabilitation treatment variables.

	All (*n* = 78)	Early CSE (*n* = 31)	Late CSE (*n* = 47)	*p* Value
Hospitalization period (days)	60 ± 27.42	57.84 ± 25.02	61.43 ± 29.07	0.617
ONS treatment as part of nutritional care %	85.9	90.3	83.0	0.511
Nutrition intervention sessions	6.01 ± 2.83	5.94 ± 2.62	6.06 ± 2.98	0.914
Swallowing rehabilitation sessions	3.85 ± 3.12	4.35 ± 3.34	0.52 ± 2.98	0.186
Day on which CSE was performed	4.6 ± 4.57	1.42 ± 0.56	6.7 ± 4.84	*p* < 0.001

**Table 3:** Nutrition status outcomes and cognitive status at discharge.

	All (*n* = 78)	Early CSE (*n* = 31)	Late CSE (*n* = 47)	*p* Value
**Body Mass Index (BMI)**	25.05 ± 5.40	26.15 ± 6.14	25.28 ± 6.08	0.950
**BMI categories (%)**				
Underweight (BMI < 18 kg/m^2^)	32.9	35.5	31.9	0.874
Normal (BMI ≥ 18–<25 kg/m^2^)	54.4	51.6	57.4	
Overweight (BMI ≥ 25 kg/m^2^)	11.4	12.9	10.6	
**MNA-SF score**	6.26 ± 2.50	6.71 ± 2.28	5.96 ± 2.62	0.160
**Nutrition status categories (%)**				
Malnutrition (score = 0–7)	71.8	61.3	78.7	0.012
At risk (score = 8–11)	23.1	38.7	12.8	
Normal (score = 12–14)	5.1	0	8.5	
**Delta (Δ) MNA-SF scores (MNA-SF score at hospital discharge minus MNA-SF score at admission)**	1.37 ± 2.13	1.97 ± 2.08	0.98 ± 2.09	0.053
**MMSE cognitive status categories (%)**				
Severe impairment (score = 0–9)	53.8	48.4	57.4	0.735
Mild/moderate impairment (score = 10–24)	23.1	32.3	17.0	
No impairment (score ≥ 25)	23.1	19.4	25.5	

MMSE = Mini Mental State Examination; MNA-SF = Mini Nutritional Assessment Short Form.

**Table 4:** (**a**) Logistic regression model of malnutrition at discharge from hospital. (**b**) Logistic regression model of malnutrition at discharge from hospital without sex or number of sessions with a dietitian.

(**a**)			
**Predictor Variable**	**Odds Ratio**	**95% Confidence Interval**	***p*-Value**
CSE timing (early = 0)	6.61	1.36–32.12	0.019
Age	1.13	1.01–1.25	0.026
Sex (male = 1)	0.61	0.14–2.69	0.509
Number of nutrition rehabilitation sessions	1.51	0.85–1.56	0.363
MMSE score at admission	2.70	1.03–7.04	0.043
MNA-SF at admission	0.52	0.34–0.79	0.003
(**b**)			
**Predictor Variable**	**Odds Ratio**	**95% Confidence Interval**	***p*-Value**
CSE timing (early = 0)	5.87	1.28–26.99	0.023
Age	1.12	1.02–1.24	0.025
MMSE score at admission	2.69	1.05–6.90	0.049
MNA-SF at admission	0.52	0.35–0.79	0.002

MMSE = Mini Mental State Examination; MNA-SF = Mini Nutritional Assessment Short Form. For analyses in Table 4a,b, the MNA-SF at the time of hospital discharge score was dichotomized into a new variable, “malnutrition,” such that an MNA-SF score of 0–7 indicated the presence of malnutrition (malnutrition = 1) and scores >7 indicated the absence of malnutrition (malnutrition = 0).

## Data Availability

Data are not publically available due to patient privacy concerns; however, they are available upon request to the corresponding author.
